# News and Notes

**Published:** 1905-03

**Authors:** 


### NEWS AND NOTES.

DR. ANITA NEWCOMB McGEE has been made a member of the United Spanish War Veterans, and is the only woman holding membership in that organization. Dr. McGee during the war with Spain was commissioned acting assistant surgeon.

THE semi-annual meeting of the Medical Society of the Missouri Valley will be held in Kansas City March 23, 1905. An invitation has been extended to the Presidents of the State Associations within the territory embraced by the Missouri Valley and to the profession in general, and an interesting and profitable meeting is expected.

THE Southern Medical College Association has elected the following officers: President, Dr. Christopher Tompkins, Richmond, Va.; Vice-President, Dr. J. C. LeGrande, Birmingham, Ala.; Secretary, Dr. G. C. Savage, Nashville, Tenn.; Executive Committee, Drs. J. A. Cain, Nashville; Charles Rosser, Dallas, Texas, and W. B. Rogers, Memphis, Tenn.

THE fifty-fourth meeting of the American Association for the Advancement of Science was held at the University of Pennsylvania, December 27-30. Besides the ten sections of the American Association, thirty affiliated scientific societies also held their annual meeting. This is the third time the association has met in Philadelphia.

IT is reported that there is a scarcity of various medicinal roots and herbs, especially those of American production, and that in consequence the market prices of those crude drugs have been decidedly raised of late. We may imagine that the activity of the synthetist is driving the herbalist out of business, though it may be that the soil has not recently yielded the usual amount of medicinal plants. Whatever may be the cause of the present deficiency, it is to be hoped that it will be remedied in another season, for we can hardly yet do without the old vegetable materia medica.

---

THE oldest medical works in existence are those of the Chinese, and date back to nearly 3,000 years B. C. Then, as now, they divided their subjects under the captions of healing, cooling, refreshing and temperate. They have everything divided into classes, and their prescriptions are classified under seven headings, as follows: (1) the great prescription; (2) the little prescription; (3) the slow prescription; (4) the quick prescription; (5) the odd prescription; (6) the even prescription; (7) the double prescription. These are applied under four special circumstances and conditions, which in their turn are classified. Fire is an agent in which they have great faith, as also they have in mineral waters.

---

PHYSICIANS in the East are planning a delightful itinerary in connection with the trip to Portland next year. It will include stops at Niagara Falls, Lake Minnetonka, Yellowstone Park, and Butte, Mon. The return trip will include stops in San Francisco, Los Angeles, Sacramento, Salt Lake City, Glenwood Springs, The Garden of the Gods, Denver and Omaha. The expense is estimated at $325 for each person. This will include all expenses except hotel bills at Portland and San Francisco. Information concerning this trip can be obtained by writing Dr. F. H. Wiggin, 55 West Thirty-sixth street, New York City.

---

THE Oncologic Hospital opened its new hospital and dispensary, for the study and treatment of cancer, at Forty-fifth and Chestnut Streets, on January 4th. The dispensary service will be held at 2 o'clock daily. The hospital can accommodate twenty-four patients at present. Dr. Boardman Reed is the attending physician. Dr. Addinell Hewson, Dr. G. Betton Massey, and Dr. Howard R. Swayne are the attending surgeons. Dr. William S. Newcomet is in charge of the X-ray department.

